# Beta 1, Beta 2 and Beta 3 Adrenergic Receptor Gene Polymorphisms in a Southeastern European Population

**DOI:** 10.3389/fgene.2018.00560

**Published:** 2018-11-28

**Authors:** Martha-Spyridoula Katsarou, Aikaterini Karathanasopoulou, Angeliki Andrianopoulou, Vasileios Desiniotis, Efthymios Tzinis, Efthimios Dimitrakis, Maria Lagiou, Evangelia Charmandari, Michael Aschner, Aristeidis M. Tsatsakis, George P. Chrousos, Nikolaos Drakoulis

**Affiliations:** ^1^Research Group of Clinical Pharmacology and Pharmacogenomics, Faculty of Pharmacy, School of Health Sciences, National and Kapodistrian University of Athens, Zografou, Greece; ^2^Department of Computer Science, University of Illinois at Urbana-Champaign, Champaign, IL, United States; ^3^Division of Endocrinology, Metabolism and Diabetes, First Department of Pediatrics, “Aghia Sophia” Children’s Hospital, National and Kapodistrian University of Athens Medical School, Athens, Greece; ^4^Department of Molecular Pharmacology, Albert Einstein College of Medicine, Bronx, NY, United States; ^5^Laboratory of Toxicology, Medical School, University of Crete, Crete, Greece

**Keywords:** single nucleotide polymorphisms, personalized medicine, pharmacogenomics, Ser49Gly, Arg389Gly, Gly16Arg, Gln27Glu, Trp64Arg

## Abstract

Genetic polymorphisms in β_1_-, β_2_- and β_3_-adrenergic receptors (β-ARs) have been associated with chronic non-communicable disorders, such as cardiovascular diseases, asthma, chronic obstructive pulmonary disease (COPD) and obesity, as well as β-agonists and antagonists response and toxicity. The purpose of this study was to determine the frequency distribution of *ADRB1* genetic variants Ser49Gly and Arg389Gly, *ADRB2* variants Gly16Arg and Gln27Glu, *ADRB3* variant Trp64Arg in a Southeastern European Caucasian (SEC) population sample and to establish a comparison with existing data from other human populations. A sample of 431 men and 590 women volunteered to participate in this genotyping analysis after anonymization and de-identification. Real Time PCR (Melting Curve Analysis) followed DNA extraction from buccal swabs and statistical analysis of the results was performed. The allele frequencies in the SEC population were Ser49 (90.3%), Arg389 (69.49%), Gly16 (61.61%), Gln27 (65.72%), and Trp64 (94.52%), while a Hardy-Weinberg Equilibrium (HWE) was detected in the population studied. Comparisons for the Ser49Gly, Gln27Glu, and Trp64Arg allele distributions demonstrated significant differences between SEC and the European group. European subgroups comparisons showed that allele distributions were similar for four of the five SNPs between SEC and Southwestern European Caucasians (SWC), while they were quite distinct from the Northwestern European Caucasians (NWC). These data underline the importance of interethnic variability of β-ARs genetic polymorphisms.

## Introduction

Beta-adrenergic receptors (β-ARs) are essential components of the sympathetic nervous system. β-ARs belong to the superfamily of G protein-coupled receptors (GPCRs) and their signaling pathway is stimulated by the endogenous catecholamines, epinephrine and norepinephrine (Brodde, 2008). Subsequently, AC promotes the formation of cAMP, which is the major modulator of intracellular events (Leineweber and Heusch, 2009; Masuo and Lambert, 2011). Long-term sympathetic activation through the Ga protein signaling pathway leads to β-AR desensitization and, thus, results in decreased receptor expression, a state known as downregulation (McGraw and Liggett, 2005). Considering the critical roles of β-ARs in various functions of the sympathetic nervous system, one would assume that genetic variations that alter these receptors’ functions affect susceptibility to several diseases as well as drug response and toxicity.

The *ADRB1* gene encodes β_1_-AR, which is the predominant β-AR in the heart, accounting for 70–80% of the cardiac β-ARs (Brodde, 2008). Only two of the twelve known SNPs of β_1_-AR are of functional importance, Ser49Gly or rs1801252 (145 A > G) and Arg389Gly or rs1801253 (1165 G > C). The Ser49 variant is the wild-type and has been linked with reduced 5-year survival in patients with heart failure, while the mutant Gly49 variant is correlated with a greater elevation of BMI over the years (Rydén et al., 2001; Kirstein and Insel, 2004; Taylor, 2007; Nonen et al., 2008). The Arg389Gly polymorphism is not considered a risk factor for cardiovascular disease, however, it seems to have a strong impact on the response of patients to therapeutic agents, with β-antagonists being more effective in wild-type homozygous Arg389 individuals (Ahles and Engelhardt, 2014).

More than 80 SNPs have been described in the coding region of *ADRB2*, with Gly16Arg or rs1042713 (46 G > A) and Gln27Glu or rs1042714 (79 C > G) being the most common (Ahles and Engelhardt, 2014; Danielewicz, 2014; Ferreira-Santos et al., 2017). Although the Gly16Arg polymorphism is mainly associated with respiratory diseases, this polymorphism does not affect the susceptibility to asthma or to COPD in the general population. The mutant Arg16 variant is associated with an enhanced response to short-term treatment with SABA, while several data analyses in children and adults have shown that Arg16 homozygous display adverse effects and deterioration of asthma symptoms after receiving a short-acting antiasthmatic β_2_-agonist (SABA) regularly (Johnson and Liggett, 2011; Ahles and Engelhardt, 2014; Kersten et al., 2016). Regarding the Gln27Glu polymorphism, there is no strong evidence supporting a relation between this polymorphism and the response to β-agonists and antagonists. However, the Glu27 variant has been considered to be protective against asthma (Thakkinstian et al., 2005; Ahles and Engelhardt, 2014).

The *ADRB3* gene encodes β_3_-AR, which is the major modulator of lipolysis and thermogenesis in adipose tissue (Coman et al., 2009). Among the ten SNPs that have been listed for β_3_-AR, Trp64Arg or rs4994 (64 Trp > Arg) has attracted the most scientific interest (Ahles and Engelhardt, 2014); the wild-type variant is Trp64 (Gjesing et al., 2007; Liu et al., 2007). Up-to-date population studies have linked Arg64 with a decreased metabolic rate, higher BMI and greater risk for abdominal obesity, as well as increased essential hypertension compared with Trp64, yet with inconclusive results (Liu et al., 2007; Kurokawa et al., 2008; Li et al., 2018).

The significant contribution of different β-ARs genetic variants to chronic non-communicable diseases and their importance in the response to β-agonists or antagonists is well established. Thus, illuminating the allele frequencies of β_1_-, β_2_- and β_3_-ARs polymorphisms might help with the diagnosis, treatment and prevention of major human disorders. This study examined the allele and genotype frequency distributions in a Southeastern European Caucasian (SEC) population sample and the results were compared with published data from other ethnic groups. The aim of this comparison was to demonstrate possible inter-ethnic variability, as well as the varying penetrance in different populations depending on the evolutionary stressors exerted in each group. To the best of our knowledge, this is the first study presenting the allele and genotype frequencies of Ser49Gly (*ADRB1*), Arg389Gly (*ADRB1*), Gly16Arg (*ADRB2*), Gln27Glu (*ADRB2*) and Trp64Arg (*ADRB3*) in a SEC population.

## Materials and Methods

A total of 1,021 individuals participated in the study and were of Caucasian ancestry. The study population consisted of 431 men and 590 women. Data originated from individuals that joined a predisposition genotyping program and volunteered to provide their genetic information for research. Inclusion criteria were gender, date of birth, and place of origin. All samples originated from Southeastern European Balkan area, mostly coming from Greece, Bulgaria, Albania, Romania and Serbia. Exclusion criteria were the Southwestern, Northwestern, and Northeastern European origin of volunteers, participation of more than one family member in the study, as well as volunteers that participated in the genotyping procedure for medical reasons. No further information on their medical history was retrieved. All subjects gave written informed consent for the use of their genetic information after de-identification and anonymization of the DNA samples, following the European Medicines Agency guidelines (EMEA/CPMP/3070/01). The study was approved by the Ethics Committee of the “ATTIKON University General Hospital,” National and Kapodistrian University of Athens, Greece.

Epithelial cells from the oral cavity of all volunteers were collected by buccal cotton swabs, followed by DNA extraction with the use of a commercial nucleic acid isolation kit (Tissue Nucleospin; Macherey-Nagel GmbH & Co., KG, Düren, Germany). The genotype of each participant was determined by Real Time Polymerase Chain Reaction, followed by melting curve analysis using the Simple Probe commercial LightSnip kit and the Light Cycler Fast DNA Master HypProbe Kit (Roche Diagnostics, Mannheim, Germany). Reactions were performed on a LightCycler480-Instrument platform (Roche-Diagnostics, Rotkreuz, Switzerland) in accordance with the manufacturer’s recommendations. The following gene and their respective variants were analyzed: Ser49Gly (*ADRB1*), Arg389Gly (*ADRB1*), Gly16Arg (*ADRB2*), Gln27Glu (*ADRB2*), and Trp64Arg (*ADRB3*).

All statistics and graphs were produced by using Python scripting language^1^. Statistical analysis was performed at a significance level of *a* = 0.05. The *p*-value, indicating the statistical significance, was calculated by the Fisher’s exact test and *p* < 0.05 was considered as statistically significant. The calculated ORs represent the odds of a certain allele to be present in the SEC population compared to the odds of the same allele to exist in another specific population. The corresponding confidence intervals (95% CI) were used to estimate the precision of the OR. It was also tested whether the investigated genotypes followed the HWE by the use of web based software (“Hardy-Weineberg Equilibrium Calculator, 2017,” n.d.). The HWE is a principle stating that the genetic variation in a population will remain constant from one generation to the next in the absence of genetic drift or evolutionary forces. It is used to measure whether the observed genotype frequencies in a population are different from the ones predicted by the equation (Relethford, 2012). The population was assumed to be in HWE for a polymorphism if *x*^2^ was less than the critical value of 3.84 (*p* = 0.05) for 2 × 2 table (1 d.f.) at a significance level *a* = 0.05. Data for the other populations derived from the Ensembl Genome Browser^2^.

## Results

Genotype frequency distributions of β_1_-, β_2_- and β_3_-AR polymorphisms in the studied SEC population are displayed in Table 1. The observed and expected distributions in all polymorphisms are in HWE, except for the Trp64Arg polymorphism. HWE could not be accurately determined for this polymorphism due to small number of volunteers carrying the mutant variant Arg64 (Table 1).

**Table 1:** Genotype frequencies of β_1_-, β_2_- and β_3_-adrenergic receptor polymorphisms in a Southeastern European Caucasian population (SEC).

	*ADRB1*		*ADRB2*		*ADRB3*
	Ser49Gly (rs1801252) (HWE = 3.726)	Arg389Gly (rs1801253) (HWE = 0.023)	Gly16Arg (rs1042713) (HWE = 1.931)	Gln27Glu (rs1042714) (HWE = 0.306)	Trp64Arg *ADRB3* (n.a.)^∗^
**Genotypes** (*n* = 1,021)	Ser:Ser (*n* = 838) 82.08%	Arg:Arg (*n* = 492) 48.19%	Gly:Gly (*n* = 377) 36.93%	Gln:Gln (*n* = 437) 42.80%	Trp:Trp (*n* = 911) 89.22%
	Ser:Gly (*n* = 168) 16.45%	Arg:Gly (*n* = 435) 42.60%	Gly:Arg (*n* = 504) 49.36%	Gln:Glu (*n* = 468) 45.84%	Trp:Arg (*n* = 108) 10.58%
	Gly:Gly (*n* = 15) 1.47%	Gly:Gly (*n* = 94) 9.21%	Arg:Arg (*n* = 140) 13.71%	Glu:Glu (*n* = 116) 11.36%	Arg:Arg (*n* = 2) 0.20%

*Genotype distributions of Ser49Gly, Arg389Gly, Gly16Arg, Gln27Glu and Trp64Arg polymorphisms in a sample of 1,021 individuals from SEC population. HWE, Hardy-Weinberg Equilibrium. ^∗^n.a, not available, (HWE could not be calculated for the Trp64Arg polymorphism due to the rare homozygous Arg64 allele).*

**FIGURE 1:**
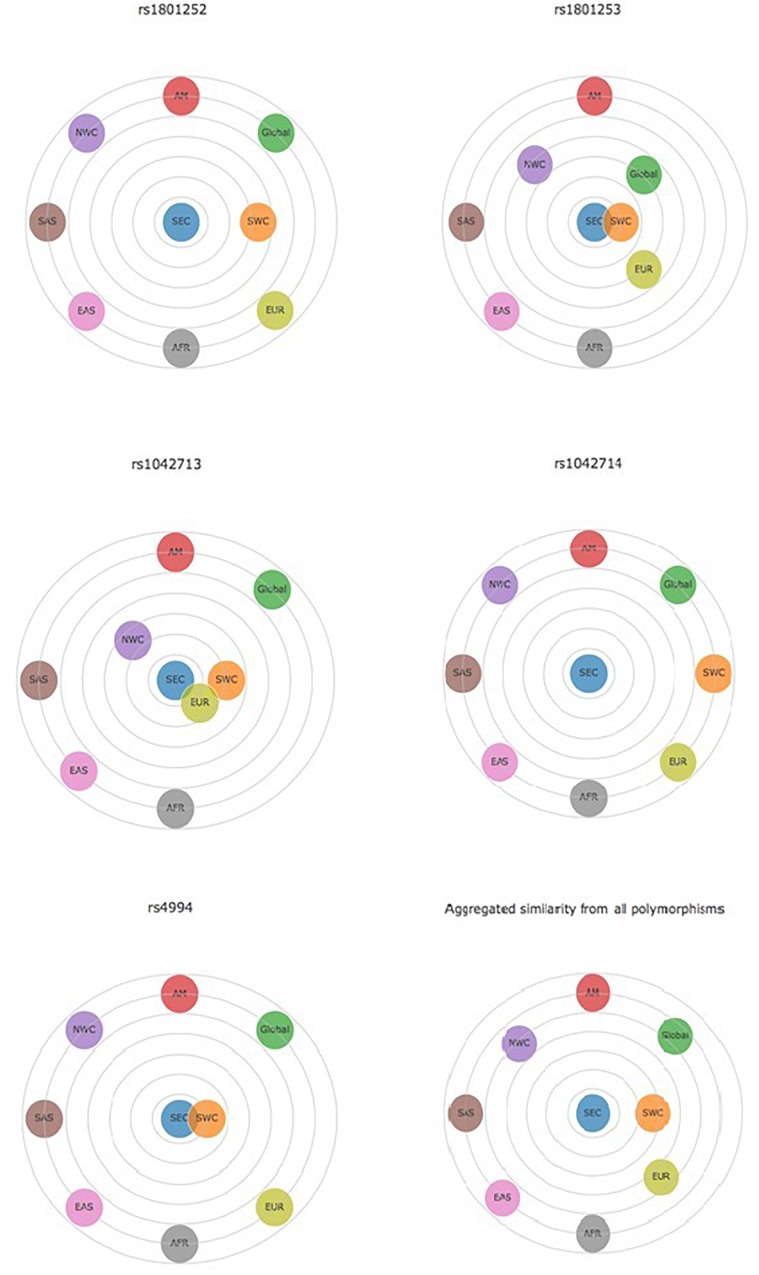
Distance graph based on *p*-value for β-adrenergic receptors polymorphisms Ser49Gly (rs1801252), Arg389Gly (rs1801253), Gly16Arg (rs1042713), Gln27Glu (rs1042714), and Trp64Arg (rs4994). All comparisons are made between SEC and other populations. Data for the other populations derived from the Ensembl Genome Browser http://www.ensembl.org/index.html. AFR, Africans; AM, Americans; EAS, East Asians; EUR, Europeans; NWC, Northwestern Caucasians; SAS, South Asians; SEC, Southeastern Caucasians; SWC, Southwestern Caucasians.

Data collected for Ser49Gly polymorphism showed that 90.30% of the volunteers carried the Ser49 allelic variant, while only 9.70% carried the Gly49 variant. The majority of the volunteers were either Ser49 homozygous (82.08%) or heterozygous (16.45%). Comparison of the SEC with other European populations revealed varying distributions between SEC and NWC, which include Northern and Western European Caucasians, Finnish in Finland and British in England and Scotland (Table 2). Differences with SWC, that include Iberian populations from Spain and Toscani in Italy, were negligible (Figure 1). Statistically significant differences were observed in allele distribution when compared with other non-European populations.

**Table 2:** Allele frequency distributions of β_1_-, β_2_-, β_3_-adrenergic receptor polymorphisms between the Southeastern European Caucasian population (SEC) and other populations.

Ser49Gly (rs1801252) (*ADRB1)*		Subjects	Ser %	Gly %	OR	95% CI	*P*
	SEC	(*n* = 1021)	90.30	9.70	–	–	–
	European	(*n* = 503)	87.28	12.72	1.36	1.07–1.72	0.0126
	NWC^1^	(*n* = 289)	85.81	14.19	1.54	1.17–2.03	0.002
	SWC^2^	(*n* = 214)	89.25	10.75	1.12	0.80–1.57	n.s.
	African	(*n* = 661)	76.10	24.90	2.93	2.41–3.55	<0.0001
	American	(*n* = 347)	74.93	25.07	3.12	2.49–3.91	<0.0001
	Asian (East)	(*n* = 504)	85.91	14.09	1.53	1.21–1.92	0.0004
	Asian (South)	(*n* = 489)	86.71	13.29	1.43	1.13–1.81	0.0033
	Global	(*n* = 2504)	82.23	17.77	2.01	1.71–2.37	<0.0001

**Arg389Gly (rs1801253) (*ADRB1)***		**Subjects**	**Arg %**	**Gly %**	**OR**	**95% CI**	***P***

	SEC	(*n* = 1021)	69.49	30.51	–	–	–
	European	(*n* = 503)	68.49	31.51	1.05	0.89–1.23	n.s.
	NWC^1^	(*n* = 289)	67.82	32.18	1.08	0.89–1.32	n.s.
	SWC^2^	(*n* = 214)	69.39	30.61	1.00	0.80–1.08	n.s.
	African	(*n* = 661)	57.03	42.97	1.72	1.49–1.98	<0.0001
	American	(*n* = 347)	80.55	19.45	0.55	0.45–0.68	<0.0001
	Asian (East)	(*n* = 504)	78.77	21.23	0.61	0.51–0.73	<0.0001
	Asian (South)	(*n* = 489)	73.42	26.58	0.82	0.70–0.98	0.0292
	Global	(*n* = 2504)	70.17	29.83	0.97	0.87–1.08	n.s.

**Gly16Arg(rs1042713) (*ADRB2)***		**Subjects**	**Gly %**	**Arg %**	**OR**	**95% CI**	***P***

	SEC	(*n* = 1021)	61.61	38.39	–	–	–
	European	(*n* = 503)	61.43	38.57	1.01	0.86–1.18	n.s.
	NWC^1^	(*n* = 289)	60.73	39.27	1.04	0.86–1.25	n.s.
	SWC^2^	(*n* = 214)	62.38	37.62	0.97	0.8–1.20	n.s.
	African	(*n* = 661)	47.96	52.04	1.74	1.51–2.00	<0.0001
	American	(*n* = 347)	54.32	45.68	1.35	1.13–1.61	0.0008
	Asian (East)	(*n* = 504)	45.14	54.86	1.95	1.67–2.27	<0.0001
	Asian (South)	(*n* = 489)	55.42	44.58	1.29	1.10–1.51	0.0013
	Global	(*n* = 2504)	52.44	47.56	1.46	1.31–1.62	<0.0001

**Gln27Glu (rs1042714) (*ADRB2)***		**Subjects**	**Gln %**	**Glu %**	**OR**	**95% CI**	***P***

	SEC	(*n* = 1021)	65.72	34.28	–	–	–
	European	(*n* = 503)	59.05	40.95	1.33	1.14–1.55	0.0004
	NWC^1^	(*n* = 289)	59.00	41.00	1.33	1.10–1.61	0.0032
	SWC^2^	(*n* = 214)	59.11	40.89	1.33	1.07–1.64	0.0105
	African	(*n* = 661)	86.38	13.62	0.30	0.25–0.36	<0.0001
	American	(*n* = 347)	75.79	24.21	0.61	0.50–0.75	<0.0001
	Asian (East)	(*n* = 504)	92.66	7.34	0.15	0.12–0.20	<0.0001
	Asian (South)	(*n* = 489)	80.67	19.33	0.46	0.38–0.55	<0.0001
	Global	(*n* = 2504)	79.57	20.43	0.49	0.44–0.55	<0.0001

**Trp64Arg(rs4994) (*ADRB3)***		**Subjects**	**Trp%**	**Arg%**	**OR**	**95% CI**	***P***

	SEC	(*n* = 1021)	94.52	5.48	–	–	–
	European	(*n* = 503)	91.85	8.15	1.53	1.12–2.05	0.0057
	NWC^1^	(*n* = 289)	89.79	10.21	1.96	1.41–2.72	<0.0001
	SWC^2^	(*n* = 214)	94.63	5.37	0.98	0.62–1.55	n.s.
	African	(*n* = 661)	90.54	9.46	1.80	1.38–2.35	<0.0001
	American	(*n* = 347)	88.04	11.96	2.34	1.74–3.15	<0.0001
	Asian (East)	(*n* = 504)	86.90	13.10	2.60	1.99–3.38	<0.0001
	Asian (South)	(*n* = 489)	84.25	15.75	3.22	2.49–4.16	<0.0001
	Global	(*n* = 2504)	88.50	11.50	2.24	1.82–2.76	<0.0001

*Comparison of allele frequency distribution (%) of β1-, β2-, β3-adrenergic receptor polymorphisms Ser49Gly, Arg389Gly, Gly16Arg, Gln27Glu, and Trp64Arg between the SEC and other populations. Data for the European, NWC, SWC, African, American, Asian (East), Asian (East), Global populations derived from the Ensembl Genome Browser http://www.ensembl.org/index.html. ^*1*^Utah residents with Northern and Western European ancestry, Finnish in Finland and British in England and Scotland, ^2^Iberian populations in Spain, Toscani in Italy, OR, Odds ratio; 95% CI, 95% confidence interval; p, p-value; n.s., non-significant differences (p > 0.05).*

The frequency of the wild-type allelic variant Arg389 was 69.49% and that of the mutant 30.51%. Of the volunteers, 48.19% were homozygous Arg389, 42.60% were heterozygous and 9.21% were homozygous Gly389. Allele distribution was quite similar between SEC and the other European populations, as well as between SEC and the global population, whereas significant differences were noted when they were compared to non-European populations (Table 2). The comparison between SEC and SWC reveals that they differ by only 0.1% (Figure 1). Americans (80.55%) and East Asians (78.77%) presented the highest frequency of the Arg389 variant, while the Gly389 allele is more frequent in Africans (42.97%) than in Europeans (31.51%).

The frequency of the wild-type Gly16 variant of Gly16Arg polymorphism was 61.61% in the subjects of the study. The majority of the volunteers were heterozygous Gly16Arg (49.36%), while 36.93% were homozygous Gly16 and 13.71% were homozygous Arg16. The Arg16 variant appeared in the lowest frequency in the SEC (38.39%) and European (38.57%) population compared to Africans, Americans and Asians (Table 2). Allele distribution of Gly16Arg in the SEC population was similar to that of the other European groups, but not to non-Europeans (Figure 1). The mutant Arg16 allele was overrepresented in Africans (52.04%) and East Asians (54.86%) compared to the wild-type Gly16. A high frequency of Arg16 variant (57%) also occurs in the African-American population (Blake et al., 2013; Ortega and Meyers, 2014).

Data obtained for the Gln27Glu polymorphism indicated a high frequency of Gln27 variant (65.72%) in the SEC population and the frequencies of homozygous Gln27 and heterozygous Gln27Glu were similar (42.80 and 45.84%, respectively). Homozygous Glu27 represented the 11.36% of the volunteers examined. The highest prevalence of Glu27 appeared in the European group (40.95%) and subgroups, with SEC presenting the lowest frequency (34.28%) among them (Table 2). This polymorphism distribution showed a distinct difference between SEC and all the others (Figure 1).

Data analysis of the Trp64Arg polymorphism demonstrated that the vast majority of the volunteers carried the wild-type allelic variant Trp64 (94.52%), while the mutant allele appeared only in a percentage of 5.48%. Homozygosity Trp64 was present in 89.22% of the volunteers, heterozygous Trp64Arg in 10.58%, while only 0.2% were homozygous for the mutant Arg64 variant (Table 1). The allele distribution of Trp64Arg polymorphism differs notably between SEC and other populations (Figure 1). When comparing SEC and NWC for the Trp64Arg polymorphism, striking differences are present (*p* < 0.0001), while the allele distributions of SEC and SWC resemble one another (Table 2).

## Discussion

SNPs in the genomic regions of β_1_-, β_2_-, and β_3_-ARs have already been associated with cardiovascular diseases, asthma, COPD and obesity in several studies. Additionally, adrenergic neurotransmission becomes functional at the early stages of brain development and interference of this transmission with receptor agonists or antagonists, many of which are commonly used in the pregnancy clinical arena, may alter brain development and function. Some of these variations are also considered to have an effect on the therapeutic response to β-agonist or antagonist treatment.

The polymorphisms Ser49Gly and Arg389Gly of β_1_-AR have been mainly correlated with lipolysis and cardiovascular diseases. Candidate gene studies have not indicated an association of Ser49Gly polymorphism with the prevalence of heart failure or hypertension (Magnusson et al., 2005; Brodde, 2008; Ahles and Engelhardt, 2014. However, this SNP might have an impact on the outcome of heart failure, as Ser49 variant is associated with reduced 5-year survival in patients undergoing β-antagonist treatment (Magnusson et al., 2005; Taylor, 2007. Research data also indicate that Gly49 carriers exhibit a greater elevation in BMI over the years.

The Arg389Gly polymorphism plays a key role in the process of signal transduction due to its position in a region within a Gs-coupling domain of the β_1_-AR (Sandilands et al., 2003; Kirstein and Insel, 2004). According to population studies, Arg389Gly polymorphism is a candidate risk factor for the development of hypertension, as homozygous Arg389 individuals display elevated heart rate and blood pressure compared to Gly389 carriers (Bengtsson et al., 2001; Brodde, 2008; Johnson and Liggett, 2011). Case-control association studies have not established a connection between the polymorphism and susceptibility to heart failure (Mialet Perez et al., 2003; Brodde, 2008). Moreover, Arg389 homozygosity has been associated with a greater improvement of hemodynamic parameters in healthy subjects, as well as decreased mortality in patients undergoing β-antagonist treatment (Sofowora et al., 2003; Bruck et al., 2005). In general, homozygous Arg389 patients seem to display a good response to β-agonist and antagonist treatment, while Gly389 carriers are considered as poor responders, who might benefit from a higher dose or a different medication (Brodde, 2008; Muthumala et al., 2008; Liu et al., 2012; Ahles and Engelhardt, 2014).

The β_2_-AR polymorphisms Gly16Arg and Gln27Glu have been investigated for their correlation with the development and treatment of respiratory diseases. Population studies demonstrated that the Gly16 variant is associated with nocturnal or severe asthma (Contopoulos-Ioannidis et al., 2005; Litonjua, 2006; Brodde, 2008). It is suggested that homozygosity for Arg16 markedly affects the development of COPD. However, a disparity in the pathogenesis of the disease exists among different subpopulations (Matheson et al., 2006). In the general population, though, the implication of Gly16Arg polymorphism in the progression of asthma and COPD, remains obscure and has not been confirmed by large clinical trials (Brøgger et al., 2006; Niu et al., 2012; Thomsen et al., 2012; Ahles and Engelhardt, 2014). Concerning the response to β_2_-agonists, Arg16 carriers are purported to exhibit a better response to one-time treatment with SABA than Gly16 carriers (Brodde, 2008; Ahles and Engelhardt, 2014). Under chronic use of SABA, Arg16 homozygous individuals might display potential drug toxicity as they are at increased risk of asthma exacerbations (Silvers and Lang, 2012; Kersten et al., 2016). Response to treatment with LABA is not considered to be genotype-dependent (Brodde, 2008; Ahles and Engelhardt, 2014). As for the Gln27Glu polymorphism, although it does not have an impact on lung function or response to β_2_-agonist treatment, the mutant Glu27 allele is thought to be protective against asthma (Taylor et al., 2000; Thakkinstian et al., 2005). β_2_-AR polymorphisms are recognized neither as contributors to heart failure and hypertension nor as predictive factors for the response to β-antagonists (Thomsen et al., 2012; Ahles and Engelhardt, 2014).

The Trp64Arg polymorphism of β_3_-AR has been significantly associated with thermogenesis and lipolysis. As reported in a large meta-analysis, the Arg64 variant contributes to susceptibility to weight gain in East Asians (Mirrakhimov et al., 2011), but this finding was not confirmed in Caucasian subjects (Kurokawa et al., 2008). Increased visceral fat mass and insulin resistance are also conferred by the presence of Arg64 variant, giving rise to the assumption that Arg64 predisposes to diabetes mellitus (Oeveren van-Dybicz et al., 2001; Gjesing et al., 2007). The Trp64Arg polymorphism has been characterized also as an independent risk factor for OAB (Qu et al., 2015; Yamamichi et al., 2015), as well as hyperuricemia, with Arg64 variant increasing serum urate levels and risk of gout (Fatima et al., 2016).

Beyond the effect of the aforementioned SNPs on predisposition and therapy of critical conditions, their functional importance lies also in the fact that they might alter drug toxicity. For instance, the adverse effects attributed to Gly16Arg polymorphism on patients receiving regular SABA is a notable example of drug toxicity due to genetic factors. Moreover, the reduced response of a genetic variant to drugs, such as the Gly389 variant to β-antagonists, might lead the physician to increase drug dose in order to have the desirable outcome. This implies a higher risk of rising the incidence of the accompanying adverse reactions as well.

The influence of the examined SNPs on the above conditions, in combination with their varying allele frequencies among populations, highlight the importance of frequency distribution analysis in different populations. As gene inter-ethnic variability is not sufficiently studied yet, purpose of the present study was to investigate whether β_1_-, β_2_- and β_3_-ARs polymorphisms distributions differ between SEC and other populations.

As presented in the results section, the comparisons between SEC and non-European populations showed significant differences for all the SNPs. This finding seems to be expectable, due to the different origins of the compared populations. Consequently, focusing on the dissimilarities between SEC and European groups attracts more interest. The allele comparison for Ser49Gly and Trp64Arg revealed significant differences between SEC and NWC but not with SWC. Concerning Arg389Gly and Gly16Arg, no important differences were detected between SEC and all the European groups, while Gln27Glu differs from all the other Europeans.

Summarizing the above, differences in three of five examined polymorphism distributions between the SEC and the European group were noted, even though both have Caucasian origin. Comparisons among European subgroups showed varying allele distributions between SEC and NWC. However, the above finding could not be replicated when comparing SEC with other SWC populations, as the majority of their distributions were roughly equal. Aggregated similarity analysis for all the polymorphisms clearly presents the above similarities and differences (Figure 1). The differences between SEC and the European group could be attributed to the fact that the latter was mostly composed of Northern Europeans. Another possible explanation for the discrepancies is the location of the examined SEC population, as Balkan countries are on the crossroads of three continents and, thus their DNA may contain residues of admixed populations. These differences might also be explained by the long-term socioeconomic and cultural influences between Southern Europeans and other non-European populations. Similar differences among south Caucasians and other Europeans have been reported in our previous studies (Katsarou et al., 2016, 2017).

Possible limitation of this study could be the small sample of the European database compared to the SEC group. More accurate conclusions about the DNA differences of these populations could be extracted from larger European samples. Despite the large size of SEC group, another weakness could be the enrollment method, as the subjects derived from a genotyping program. Thus, a truly random selection from the Balkan area might ensure a better characterization of the polymorphisms in the population studied.

## Conclusion

In conclusion, β-AR polymorphisms seem to be future candidate biomarkers for predisposition, diagnosis and therapy of crucial diseases. There is evidence that geographical locations and socioeconomic interactions among the populations might contribute to a redistribution of these SNPs. It could be assumed that influences from admixed populations also exist in other European groups sharing their borders with neighbor continents and it is important that future studies take into consideration these inter-European differences. Consequently, it is suggested that gene related studies either analyze each European group separately, or include samples from all the European subgroups in order to extract accurate conclusions. In any case, genotyping for these SNPs would be more advantageous in a personalized basis in order to overcome interethnic gene varieties, especially in populations with strong geographically dependent influences, such as SEC.

## Availability of Data and Materials

The datasets analyzed during the current study are available from the corresponding author on reasonable request and the source code used is available at https://github.com/etzinis/polymorphisms_statistic_analysis.

## Author Contributions

AK, AA, and VD wrote the manuscript. MS-K and ND designed the research. AK, AA, and ML performed the research. ET analyzed the data. ED, EC, GC, AT, and MA reviewed corrections. All authors read and approved the final manuscript.

## Conflict of Interest Statement

The authors declare that the research was conducted in the absence of any commercial or financial relationships that could be construed as a potential conflict of interest.

## Footnotes

1 https://github.com/etzinis/polymorphisms_statistic_analysis

2 http://www.ensembl.org/index.html

## Abbreviations

AC: Adenylyl cyclase
ADRB: β-Adrenergic receptor gene
AR: Adrenergic receptor
BMI: Body mass index
cAMP: cyclic AMP
CI: Confidence Intervals
COPD: Chronic Obstructive Pulmonary Disease
Gi: Inhibitory G protein
Gs: Stimulatory G protein
HWE: Hardy-Weinberg Equilibrium
LABA: Long-acting β_2_-agonists
NWC: Northwestern European Caucasians
OAB: Overactive bladder syndrome
OR: Odds ratio
SABA: Short-acting β_2_-agonists
SEC: Southeastern European Caucasians
SNP: Single Nucleotide Polymorphism
SWC: Southwestern European Caucasians.
